# The Cortical Step Sign Fails to Prevent Malrotation of a Nailed Femoral Shaft Fracture: A Case Report

**DOI:** 10.1155/2014/301723

**Published:** 2014-01-29

**Authors:** Taranjit Tung, Ted Tufescu

**Affiliations:** ^1^Division of Orthopaedic Surgery, Boundary Trails Health Centre, P.O. Box 2000, Station Main, Winkler, MB, Canada R6W 1H8; ^2^Division of Orthopaedic Surgery, Health Sciences Centre, University of Manitoba, AD420–820 Sherbrook Street, Winnipeg, MB, Canada R3A 1R9

## Abstract

Intramedullary nailing has become the treatment of choice for diaphyseal femur fractures. Malrotation is a well-recognized complication of femoral nailing. Various techniques including the cortical step sign (CSS) have been described to minimize iatrogenic rotational deformity during femoral nailing. We present a case in which the use of the CSS resulted in a clinically significant malrotation requiring revision.

## 1. Introduction

Incidence of clinically significant rotational malalignment (≥15 degrees) [1, 2] following intramedullary nailing of femoral shaft fractures is reported in up to 28% of cases [3]. To minimize this iatrogenic rotational deformity, various techniques have been described to assess and correct intraoperative femoral rotation during intramedullary fixation of femur fractures [4–8]. More recently, the significance of the cortical step sign (CSS) in assessing femoral rotation in noncomminuted femur fractures was analyzed in cadaveric specimens [9]. Although avoidance of the CSS in comminuted femur fractures is recommended, its use in cases where one cortex is noncomminuted, as in Winquist and Hansen type II fractures, is not known. We present a case in which the CSS failed to detect a 15° malrotation during antegrade nailing of one such fracture.

The patient has provided informed written consent for print and electronic publication of this case report.

## 2. Case Report

**Figure 1:**
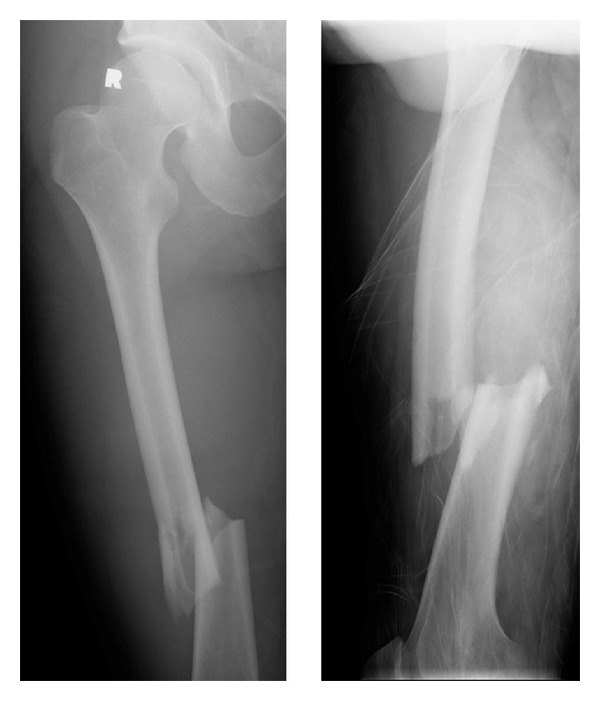
Preoperative images of the right diaphyseal Winquist and Hansen type II femur fracture. Note the lateral butterfly fragment, and the relatively transverse fracture through the medial cortex.

This is the case of an 18-year-old male who was ejected from his Ski-Doo when it collided with a rock. He presented to our facility with an isolated, closed, right diaphyseal femur fracture. The fracture had a small lateral butterfly fragment, with otherwise a simple transverse fracture through the medial cortex, Winquist and Hansen Type II fracture (Figure 1) [10]. After an informed consent the patient was positioned supine on a radiolucent operating table for intramedullary nailing of his femur. Reduction was acquired with manual traction. An antegrade reamed nail (Synthes, Mississauga, Ontario) with a piriformis start point was inserted. The nail was locked proximally. The cortical step sign (CSS), using the non-comminuted medial cortex, was employed to assess intraoperative femoral rotation. When the medial cortical widths of the proximal and distal fragments were noted to be equal on an anteroposterior (AP) fluoroscopic image, the intramedullary nail was locked distally using the free-hand technique.

**Figure 2:**
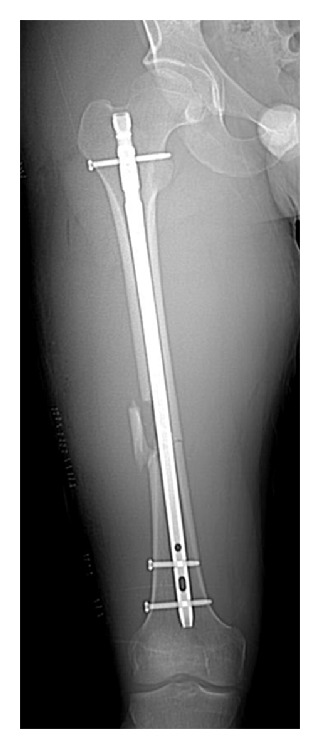
Anteroposterior CT scout image of the right femur. The cortical widths of the medial proximal and distal fragments appear to be equal.

**Figure 3:**
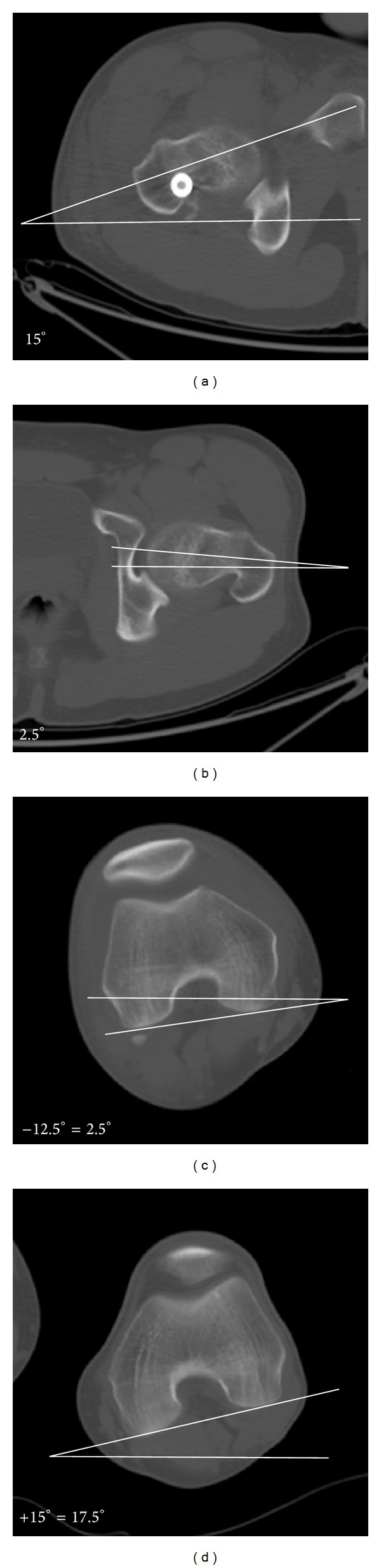
Axial cuts through femoral neck and condyles. Modified Jeanmart technique revealed 15° of external rotation of the right femur relative to the unaffected left femur.

**Figure 4:**
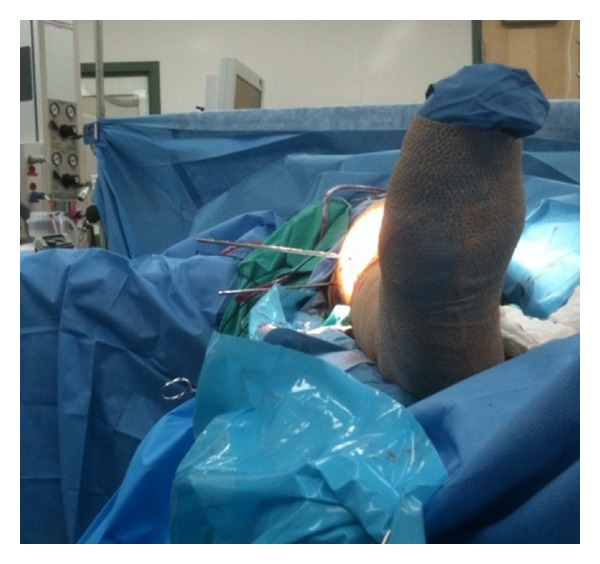
Intraoperative photograph illustrating the position of the Steinmann pins to achieve the necessary rotational correction.

**Figure 5:**
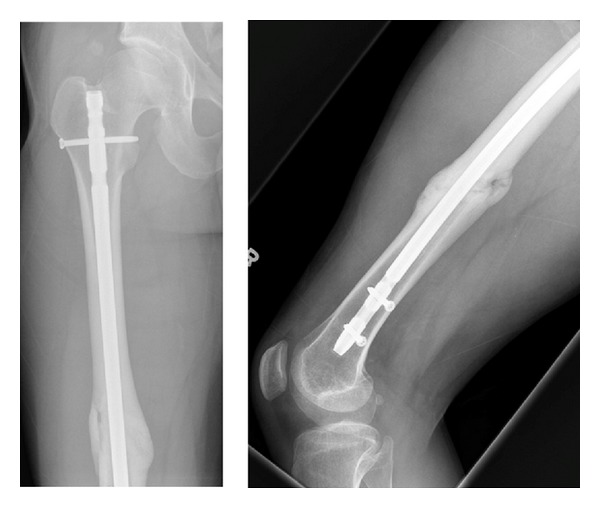
Anteroposterior and lateral radiographs illustrating adequate callus formation at the fracture site at time of final followup.

On postoperative day one the patient had adequate pain control, and his distal neurovascular examination was normal. His only concern was that he felt that his right foot was externally rotated when mobilizing. On examination in the supine position, a clinically detectable external rotation of his right lower extremity was noted. A CT scan of his lower extremities was obtained to measure the femoral rotation deformity. On the antero-posterior scout CT image the cortical widths of the medial proximal and distal fragments were equal (Figure 2). However, applying the technique described by Jeanmart et al. [11] to the axial cuts of the same CT scan, we measured a rotational deformity of 15° external rotation for the right femur (Figure 3). This finding and its implications were discussed with the patient. The patient opted for a revision surgery. Once again, the patient was positioned supine on a radiolucent operating table. One Steinmann pin was inserted into each of the lateral femoral cortises of both the proximal and distal fragments. In the axial plane, the distal Steinmann pin was inserted in 15° of external rotation relative to the proximal one (Figure 4). The two distal locking screws were removed. The right leg was then internally rotated until the pins were parallel in the axial plane. At that point the intramedullary nail was locked distally using the free-hand technique. Postoperatively the patient was satisfied with the rotation of the right leg. He made an uncomplicated recovery thereafter (Figure 5).

## 3. Discussion

Intramedullary nailing is the treatment of choice for diaphyseal femur fractures in the adult population [10]. Smaller incisions with less soft tissue damage, high union rates, and early mobilization are but a few benefits of this technique over the traditional plate and screw systems. However, as the fracture site is not evaluated under direct visualization intraoperatively, rotational malunions may occur. Incidence of clinically significant rotational malalignment (≥15°) [1, 2] following intramedullary fixation of femoral shaft fractures has been reported to be up to 28% [3]. To minimize iatrogenic rotational deformity, various clinical and radiographic techniques have been described to assess and correct intraoperative femoral rotation during intramedullary fixation of femur fractures [4–8]. One such radiographic technique is the cortical step sign (CSS) [8]. This technique involves comparing the cortical widths of the proximal fragment to those of the distal one. Once the fracture is reduced, any discrepancy in the widths is attributed to rotational malalignment. A recent anatomic and radiographic study by Langer et al. attempted to critically analyse the concept of the CSS [9]. In their study they osteotomized 1 cm sections of diaphyseal bone from proximal, middle, and distal cadaveric femurs, and evaluated the changes in cortical widths upon rotation within a 60° arc of motion. For the proximal and middle segments both internal and external rotations were noted to decrease medial and lateral cortical widths in 70%–100% of the specimens (up to 2.2 mm, or a 21% change in cortical width). For each 10° rotation the medial cortical width changed by a greater proportion compared to the lateral cortical width. This study suggested that the CSS is a reliable tool to assess femoral rotation when both cortices are non-comminuted. Although avoidance of the CSS in comminuted femur fractures is recommended, its use in cases where one cortex is non-comminuted, as in Winquist and Hansen type II, is not known. Given that the medial cortex showed the greatest amount of change in width upon rotation in Langer et al.'s study, it begs the question whether the CSS can still be used to assess intraoperative femoral rotation in cases with a non-comminuted medial cortex, irrespective of lateral comminution. Our case illustrates that in spite of perfect radiographic cortical widths of the medial proximal and distal fragments, postoperatively the patient had a clinically measurable external rotation deformity. CT axial cuts revealed a 15° external rotation deformity of the operative extremity, while the accompanying CT AP scout image reveals no CSS. Assessing femoral rotation based purely on clinical exam such as matching the soft tissue tension and skin fold geometry to that of the unaffected limb has been associated with a higher rate of malrotation 12.46° (range 6.4°–17.7°), when compared with using a fluoroscopy assisted technique 4.1° (range 0°–9.9°) [6]. Thus various fluoroscopic techniques have been described to help correct intraoperative femoral malrotation. The Deshmukh's technique [6] that relies on the profile of the contralateral normal limb's lesser trochanter with the knee in neutral position as a reference and the Tornetta's technique [4] that uses the contralateral normal limb's femoral version as a reference are but two such fluoroscopic techniques described in the literature to help avoid iatrogenic intraoperative malrotation when nailing femoral fractures. Early results of more recently described computer assisted techniques to help prevent intraoperative femoral malrotation also look promising [12] and may offer an additional or an alternative tool in selected facilities where this technology is available.

In summary, this case highlights the limitation of the CSS in evaluating femoral rotation when only one cortex is available for assessment. Eccentric reaming of the proximal non-comminuted medial cortex is one hypothesis to explain this phenomenon. In cases where only one cortex is available for assessment, maintenance of fracture reduction during reaming and use of alternative techniques to assess femoral rotation is recommended.
